# Biological age prediction using a DNN model based on pathways of steroidogenesis

**DOI:** 10.1126/sciadv.adt2624

**Published:** 2025-03-14

**Authors:** Qiuyi Wang, Zi Wang, Kenji Mizuguchi, Toshifumi Takao

**Affiliations:** Institute for Protein Research, Osaka University, Osaka 565-0871, Japan.

## Abstract

Aging involves the progressive accumulation of cellular damage, leading to systemic decline and age-related diseases. Despite advances in medicine, accurately predicting biological age (BA) remains challenging due to the complexity of aging processes and the limitations of current models. This study introduces a method for predicting BA using a deep neural network (DNN) based on pathways of steroidogenesis. We analyzed 22 steroids from 148 serum samples of individuals aged 20 to 73, using 98 samples for model training and 50 for validation. Our model reflects the often-overlooked fact that aging heterogeneity expands over time and uncovers sex-specific variations in steroidogenesis. This study leveraged key markers, including cortisol (COL), which underscore the role of stress-related and sex-specific steroids in aging. The resulting model establishes a biologically meaningful and robust framework for predicting BA across diverse datasets, offering fresh insights and supporting more targeted strategies in aging research and disease management.

## Teaser

A DNN model reveals how sex-specific steroid variations influence aging, offering fresh insights into biological age prediction.

## INTRODUCTION

Aging is a complex and inevitable process involving the accumulation of cellular and molecular damage, leading to functional decline and an increased risk of age-related diseases (*1*, *2*). Conditions such as Alzheimer’s disease, Parkinson’s disease, and osteoporosis are closely tied to aging and substantially contribute to the health challenges faced by the elderly (*2*–*6*). Despite medical advancements, these diseases remain incurable, with current strategies focused on slowing their progression through early diagnosis and management (*7*–*10*). Accurately assessing an individual’s biological age (BA), which reflects their physiological state, is essential for understanding aging and developing effective interventions. Unlike chronological age (CA), which simply measures the passage of time, BA provides insights into the biological processes underlying aging (*11*). However, determining BA is complex, as it is influenced by both genetic and nongenetic factors, and no universally accepted standards for BA measurement currently exist (*12*–*17*). Early methods, which used phenotypic indicators like lung capacity and grip strength, lacked precision and standardization, limiting their predictive utility for aging-related diseases (*18*–*23*).

In recent years, researchers have shifted from surface-level indicators to more intrinsic measures that better capture physiological aging. Common diagnostic tools like complete blood counts and biochemical tests are frequently used to model BA, offering valuable insights into an individual’s health (*24*). However, these markers often fail to provide a direct window into the specific physiological or metabolic pathways that drive aging. To address this, omics technologies—such as genomics, epigenomics, transcriptomics, proteomics, and metabolomics—have been used to analyze aging at a molecular level (*25*). These approaches generate high-dimensional data, revealing complex interactions among potential biomarkers. Given the vital role of nongenetic factors in aging, methods like epigenomics and metabolomics, which are sensitive to environmental and lifestyle influences, have proven particularly effective in enhancing the accuracy of BA models (*12*, *13*, *26*). Building on these advancements, steroid hormones have emerged as crucial indicators of physiological aging due to their regulation of key metabolic processes (*27*–*30*). Stress-related corticosteroids and sex hormones, both of which strongly correlate with aging, present a promising avenue for refining BA predictions. Steroid profiles not only complement traditional biomarkers like DNA methylation but also offer a data-driven lens into the biological heterogeneity of aging, including sex-specific differences. By incorporating biological relationship between these hormones into BA models, it becomes possible to more accurately reflect the underlying physiological state of aging individuals.

Developing precise BA models has become a central focus in bioinformatics, with researchers using various biomarkers to estimate BA (*31*). Methods such as least absolute shrinkage and selection operator (LASSO) and Ridge regression have been applied to DNA methylation and proteomics data (*32*–*34*). While these methods are effective at identifying linear relationships, they often overlook the biomarkers linked to metabolic pathways, which are critical to understanding aging (*35*, *36*). Traditional machine learning techniques, though useful for preventing overfitting and balancing model complexity, struggle to capture the nonlinear interactions inherent in biological systems (*12*). As a result, these methods may miss the intricate biological processes underlying aging and fail to account for the substantial impact of environmental and lifestyle factors.

To overcome these challenges, modern machine learning techniques—such as support vector machines (SVMs) (*37*, *38*), random forests (*39*), and deep neural networks (DNNs) (*17*, *40*, *41*)—have gained prominence. These approaches excel at modeling nonlinear relationships, making them particularly well suited for capturing the complex biological processes involved in aging. DNNs, in particular, are effective at handling high-dimensional data and are widely used for tasks such as predicting BA. Researchers, including Levine (*17*), Mamoshina *et al*. (*40*), and Putin *et al*. (*41*), have used public datasets of blood tests and biochemical markers to predict BA using DNNs, leveraging their capacity for feature learning. Mamoshina *et al*. (*42*) also applied similar techniques to gene expression data from muscle samples, identifying age-related markers. Despite their fitting capabilities, however, DNNs are prone to overfitting, especially when involving numerous hidden layers and nodes, which can reduce performance on unseen data (*43*–*45*). Regularization techniques, cross-validation, and data augmentation are typically used to mitigate these issues, but challenges remain. One major limitation of current BA models is their emphasis on minimizing prediction errors—often measured by mean absolute error (MAE) or mean squared error (MSE)—which may not fully capture the increasing heterogeneity of aging over time (*46*, *47*). Additionally, DNNs often function as “black boxes,” making it difficult to derive biological meaning from the learned features. To uncover meaningful aging mechanisms, it is essential to address the biological interpretability of BA models, particularly in integrating biologically meaningful pathways such as steroidogenesis.

**Fig. 1.**
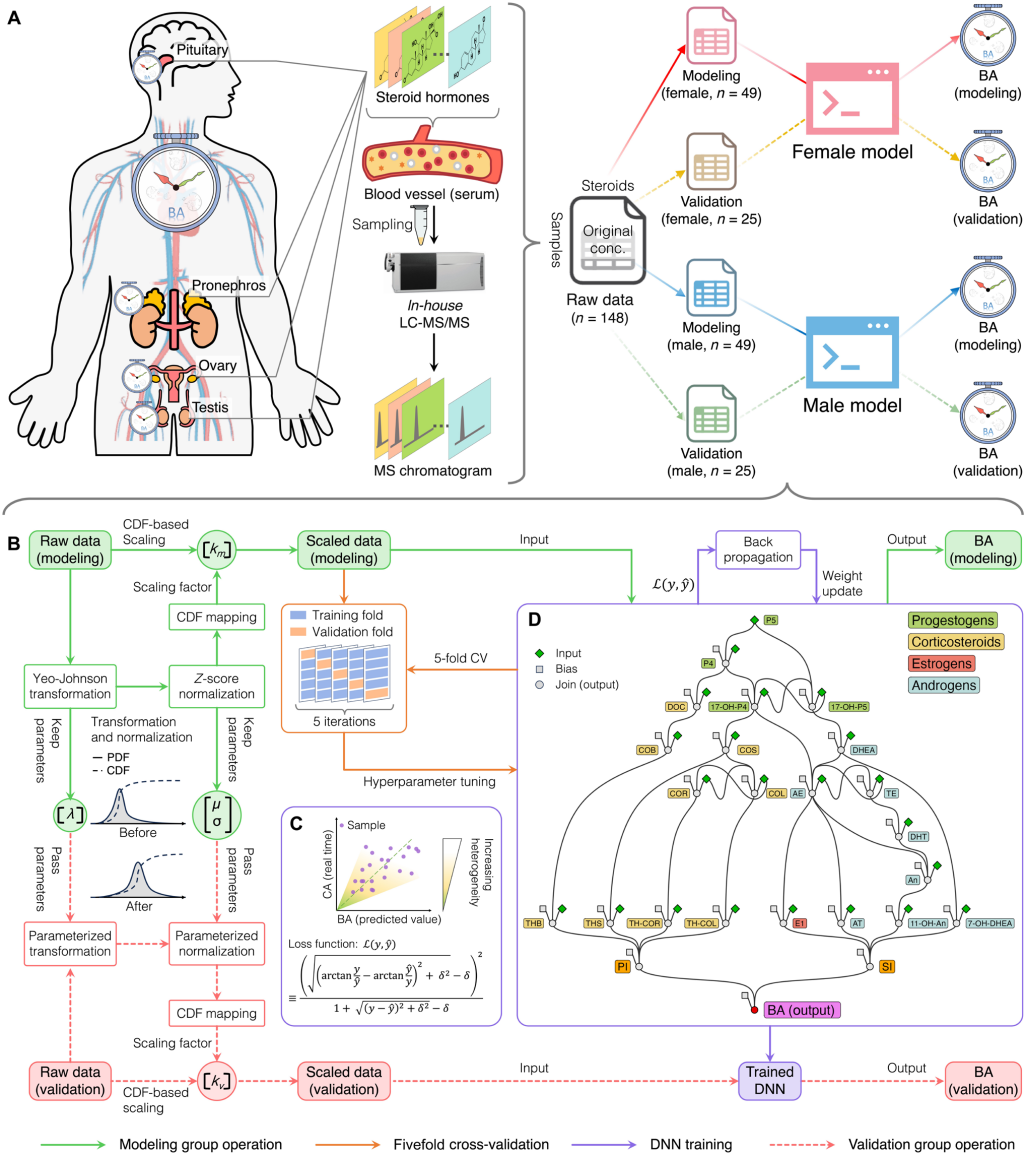
Pathway-based DNN model for BA prediction from serum steroid profiling via LC-MS/MS. (**A**) Steroid hormone quantification in blood using the LC-MS/MS method, with data divided into four groups by sex and assigned to either training with 98 samples or validation with 50 samples. (**B**) Tailored data scaling techniques were applied to address physiological and experimental variability, ensuring a reliable training dataset. This process included a CDF-based proportional scaling method, Yeo-Johnson transformation, and *z*-score normalization to maintain relative steroid concentration differences across samples. (**C**) The DNN model reflects aging heterogeneity and lifestyle-related variations by integrating known steroid pathways. A custom WSATL function balances prediction accuracy by weighting differences between BAs and CAs, preventing overfitting. (**D**) The DNN model identified key steroid pathways associated with aging, enhancing biological interpretability for BA prediction. The framework, based on steroid metabolic pathways extracted from the KEGG database, consists of 25 nodes connected by 37 directed edges, revealing the steroidogenesis that influences aging.

Here, we developed a DNN model centered on pathways of steroidogenesis to enhance the accuracy of BA prediction. Steroids were quantified using an in-house liquid chromatography–tandem mass spectrometry (LC-MS/MS) method (*48*), with the resulting data stratified into four groups according to sex and designation for training or independent validation (Fig. 1A). To address physiological and experimental variability, we applied tailored data scaling techniques that preserved the inherent relative proportions of steroid concentrations while achieving reliable alignment between the training and validation datasets (Fig. 1B). The DNN model also incorporates a custom-designed loss function, specifically constructed to account for the progressive heterogeneity of aging—a feature largely neglected in previous predictive models (Fig. 1C). Furthermore, the DNN architecture was structured to capture biochemical process within key steroid pathways, thus substantially enhancing the model’s biological interpretability (Fig. 1D). Through consideration of sex-specific steroidogenesis and validation with independent datasets, our goal was to establish a DNN-based BA prediction model that effectively represents diverse aging patterns across populations and reflects fundamental biological pathways.

## RESULTS

### DNN dataset generation via steroid quantification using LC-MS/MS

We applied a previously established method to quantify 30 steroid hormones in serum, with the list of compounds and their structures shown in table S1 and fig. S1 and the experimental parameters outlined in table S2. Validation results, including assessments of limit of quantitation (LOQ), linearity, recovery, precision, and accuracy (table S3), confirm the method’s robustness for steroid quantification.

We used this validated method to quantify 22 steroids in 150 individuals, aged 20 to 73, with detailed results presented in table S4. Out of the 100 samples used for modeling, two were excluded due to one exceeding the maximum LOQ and the other having most compounds below the LOQ, while 50 samples were used for validation (Fig. 1A). As shown in fig. S2 and table S5, the concentrations varied widely but mostly aligned with previous studies. Differences in estrone (E1) levels in female samples likely stemmed from menstrual cycle variations. The broader range of 7α-hydroxydehydroepiandrosterone (7-OH-DHEA) in our study may reflect the inclusion of younger, more diverse participants, whereas previous research focused on older individuals (aged 50 to 91). Comparisons with previous studies on tetrahydrocortisol (TH-COL), tetrahydrocortisone (TH-COR), 11-β-hydroxyandrosterone (11-OH-An), tetrahydrocorticosterone (THB), tetrahydrodeoxycortisol (THS), and adrenosterone (AT) are limited due to smaller sample sizes in those studies (fewer than 20 subjects), while our dataset is more robust (see table S6).

To conduct DNN modeling, we gathered additional demographic and physiological information for each individual subject, including CA, sex, ethnicity, ABO blood types, Rh blood types, and smoking habits (applicable only to the independent validation datasets). Recognizing the potential influence of sex-specific factors on steroid concentrations, we performed principal components analysis (PCA) on the original modeling dataset to assess the need for sex-specific models. As shown in fig. S3, PCA revealed a clear separation between sexes along PC2 (fig. S3A), which accounted for 14.7% of the variance (fig. S3B). This separation was primarily driven by specific steroids such as P5, 11-OH-An, An, DOC, E1, P4, DHT, TE, and AE—most of which are sex hormones (fig. S3, C and D). Furthermore, distinct correlations between CA and higher principal components (PC3) were observed in female and male groups (fig. S3E), underscoring inherent biological differences in steroid profiles between sexes and supporting the need for separate sex-specific models. For downstream analysis, the female and male datasets were further divided into 49 samples for training and 25 for independent validation (Fig. 1A), ensuring robust model evaluation and biological relevance.

Additionally, PC1 accounted for 32.1% of the total variance, and its factor loadings revealed a strong positive correlation among steroid variables, suggesting a coherent pattern across individuals, likely reflecting shared biological rhythms (fig. S3C). To further investigate this synchronization, we conducted an interindividual correlation analysis using the raw concentrations of 22 measured steroids (fig. S4A). The analysis revealed an average correlation exceeding 98% across individuals, consistent across all groups (fig. S4B). These findings suggest that while interindividual synchronization dominates, subtle variability remains present and must be accounted for. To address this, we implemented a scaling approach designed to preserve biological consistency while minimizing batch effects for downstream modeling.

### Maintaining biological consistency and minimizing batch variability

Building on the initial observations of raw steroid concentration data, we implemented a cumulative distribution function (CDF)–based proportional scaling method to refine the dataset, aiming to preserve inherent relative proportions of steroid concentrations while reducing batch variability across samples. This approach transforms each sample’s steroid concentrations by aligning them with a reference distribution, facilitating consistent downstream modeling. Specifically, the scaling process began with a Yeo-Johnson transformation followed by *z*-score normalization to approximate normality, standardizing across variables while retaining relative concentration differences.

**Fig. 2.**
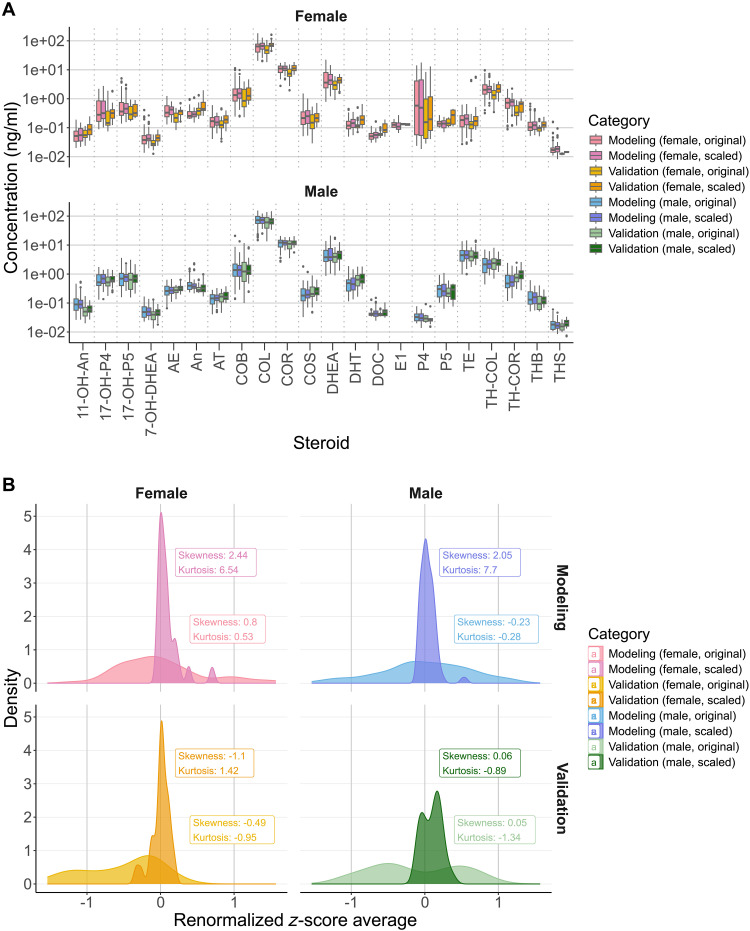
Impact of CDF-based proportional scaling on steroid concentration distributions. (**A**) Steroid concentration distributions before and after scaling for modeling and validation datasets, stratified by sex. (**B**) Density distributions of *z* scores for original and scaled steroid concentrations across modeling and validation datasets by sex.

The similarity in distribution patterns for scaled concentrations in both the modeling and validation datasets indicates that scaling effectively preserves the relative proportions of steroid concentrations within each sample (Fig. 2A). Notably, proportional differences among steroids are maintained, with reduced intergroup variability, as shown by the closer alignment of concentration distributions across cohorts compared to the original data.

To further evaluate scaling’s impact on overall sample distribution, we renormalized the scaled steroid concentrations into *z* scores for each individual and averaged these values across all steroids. This was then compared with *z* scores derived from the original, unscaled steroid concentrations to assess cumulative profiles (Fig. 2B). In both the modeling and validation groups, the distribution shifted from a broad, flat pattern in the original data to a more concentrated distribution centered around a *z* score of zero, indicating reduced sample-to-sample variability. This outcome suggests that scaling effectively aligns individual distributions and minimizes batch effects while preserving biologically relevant concentration gradients between steroids.

Moreover, analysis of the scaled steroid concentration distributions in the modeling dataset showed no statistically significant differences across ABO blood types (fig. S5A), Rh blood types (fig. S5B), or ethnicities (fig. S6), further supporting the robustness of the scaling method. This finding suggests that these demographic labels are unlikely to influence subsequent modeling outcomes. Together, these results underscore the dual advantages of this CDF-based proportional scaling approach: preserving essential steroid concentration patterns and minimizing biases that could otherwise compromise model accuracy and generalizability. The enhanced uniformity across samples and consistency in relative proportions of steroid concentrations within the scaled data are expected to strengthen model robustness, ensuring that input data accurately reflect biologically relevant variation.

### DNN design: Unveiling pathway biological features and sex-specific insights

Building on the achieved uniformity and minimized batch variability in scaled steroid concentrations across demographic subgroups, we implemented our metabolic pathway–based DNN to predict BA. The model’s architecture is explicitly designed to reflect the sequential stages of steroid biosynthesis: starting from pregnenolone (P5) as the precursor, progressing through intermediate metabolites, and culminating with the physiological indices, pressure index (PI) and sexual index (SI), along with the final BA prediction. This structured design enables the DNN to model pathways of steroidogenesis, including both active steroids and their downstream excretory metabolites, which are known to influence aging, particularly under various hormonal conditions relating with stress and sex.

To embed biologically meaningful pathways, we initialized the edge weights according to Spearman correlations among steroids and between steroids and CA (fig. S7). This initialization avoids random weights—a common source of instability in DNN models—and reduces biases linked to irrelevant biological processes by uniformly setting initial bias values to zero. To capture the heterogeneity of aging across CA, we used a custom weighted symmetric arc-tangent loss (WSATL) function, which penalizes disproportionate predictions and maintains symmetry in the model’s handling of high and low biases across different CA ranges. In contrast to conventional DNN approaches, which may misinterpret heterogeneity as prediction noise or instability, our method intentionally integrates this variability as a biologically meaningful signal. By capturing the increasing variance between predicted BA and CA over time, our model aligns with observations from aging studies and provides insights into the intricate biological complexity often overlooked in traditional frameworks.

Training optimization for enhancing model robustness and reducing the risk of overfitting was conducted through fivefold cross-validation to select hyperparameters, specifically the learning rate (*lr*) and number of epochs (*t*), ensuring a balance between validation fold loss, training stability, and computational efficiency (fig. S8). On the basis of these evaluations, a learning rate of 0.005 with 4000 epochs was selected for females, and a learning rate of 0.003 with 8000 epochs was selected for males, yielding smooth convergence with minimal loss fluctuation across iterations and contributing to stable training dynamics (fig. S9, A and B). The scatter distribution of predicted BA against actual CA reflects the intended design of the loss function, illustrating that the heterogeneity of aging expands over time and that both overestimations and underestimations across various CA segments are balanced (fig. S9C).

**Fig. 3.**
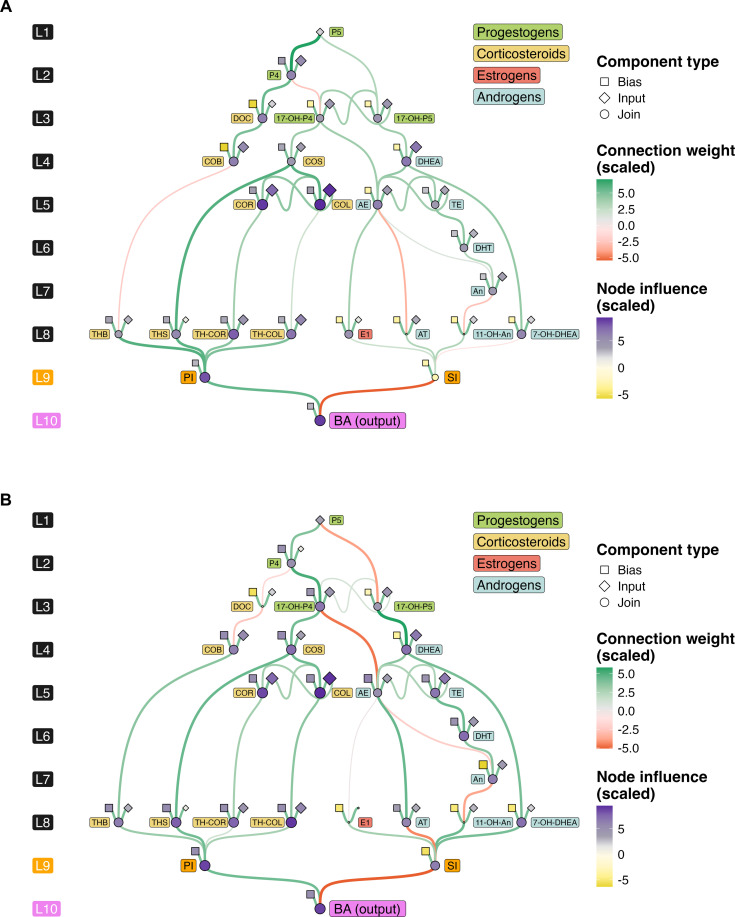
Visualization of the DNN model constructed on pathways of steroidogenesis. Sex-specific variations in steroid pathways for female (**A**) and male (**B**) models. Distinct colors are used to represent different steroid classes in the steroid labels. Connection weights reflect the influence of hierarchical steroidogenic pathways on BA prediction. Node influence reflects the average contribution of each node as it propagates through the pathway network. Component types illustrate the various sources of endogenous and exogenous influences. Detailed edge weight values and node values can be found in table S7. Bias, contribution from external pathways; Input, initial concentration; Join, summarized contributions from upstream metabolites.

The final trained models, depicted in Fig. 3A (female) and Fig. 3B (male), illustrate the pathways of steroidogenesis, highlighting the nodes and connections with the greatest impact on BA predictions (table S7). Visualization of the scaled weights between pathway components (connection weights) enables the identification of a hierarchy of steroid influence on BA prediction. The average contribution of each node (node influence) propagates through the pathway network, providing an indication of each node’s relative role in predicting BA. Additionally, categorizing nodes by origin (component type) allows for the differentiation of endogenous and exogenous influences, offering insights into their respective contributions within the model.

Notably, corticosteroid and sex hormone pathways markedly contribute to BA, with distinct impacts observed between female and male models, consistent with physiological differences. Corticosteroid nodes show strong positive associations with PI in both models, aligning with established research linking elevated corticosteroids to stress-related aging effects and supporting the hypothesis that stress pathways play a substantial role in aging across sexes. The DNN also captured sex-specific patterns within the steroid pathway: Estrogen-related nodes, such as the E1 join node, exhibited heightened influence in the female model, while androgen-related nodes, such as the AT join node, were more pronounced in the male model. This finding emphasizes the physiological specificity embedded within the DNN, in line with sex-specific hormonal profiles and their aging implications.

These pathway-based DNN models offer a robust, biologically informed approach to predicting BA by precisely leveraging steroidogenesis. To validate the DNN models identified, we assessed their predictive accuracy and generalizability, particularly in capturing BA variability across diverse independent validation datasets.

### DNN model performance and smoking impact on BA prediction

**Fig. 4.**
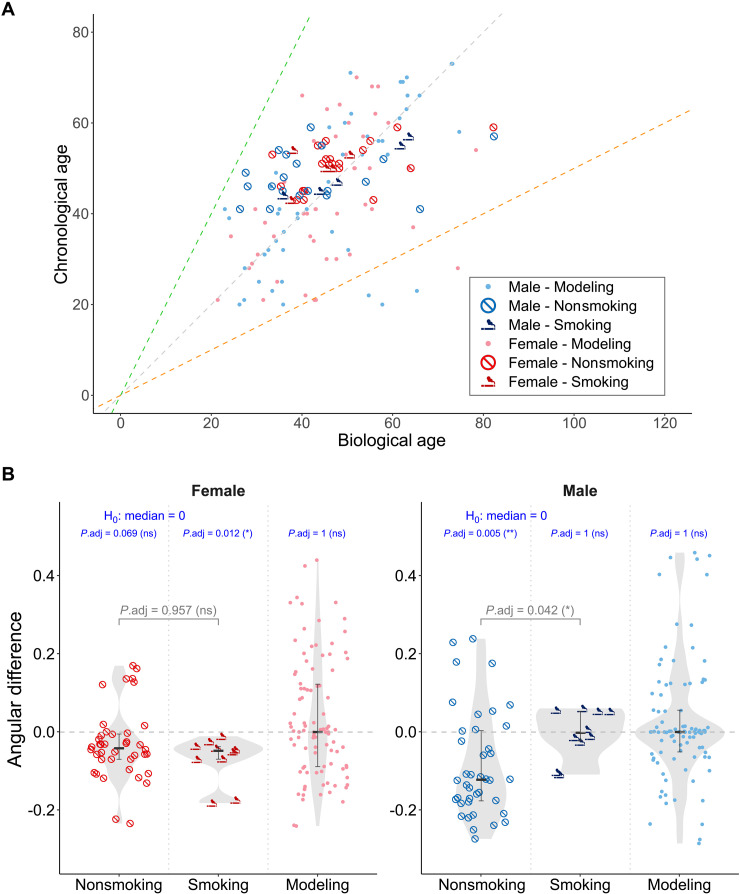
Performance of the DNN model and smoking impact on BA prediction. (**A**) Scatter plot of predicted BA versus CA for modeling and validation samples, including both smoking and nonsmoking groups. The dashed lines represent the boundaries of the twofold change, which can be interpreted as physiological thresholds indicative of a younger or older biological state. (**B**) Statistical analysis of angular differences for female and male samples. The gray *P* values indicate the differences between the smoking and nonsmoking groups, while the navy values represent the *P* values for each group in relation to the null hypothesis (H_0_) set to a median value of zero. Statistical comparisons were performed using the Wilcoxon test adjusted by Bonferroni correction. The groups include nonsmoking (validation, *n* = 40), smoking (validation, *n* = 10) for each sex, and modeling (smoking status unknown; female, *n* = 48; male, *n* = 49) individuals. ns *P*_adj_ ≥ 0.05; *P_adj_ < 0.05; **P_adj_ < 0.01.

To assess the performance of the established DNN model, we analyzed the scatter distribution of predicted BA against actual CA for both the model training group and the independent validation group. The distribution of individual prediction results suggests that the boundaries of a twofold change can be interpreted as physiological thresholds indicative of a younger or older biological state. Notably, most predictions fall within this twofold change range (Fig. 4A). Statistical analysis of the WSATL value across the various groups reveals no significant differences in prediction losses, indicating consistent performance across the cohorts (fig. S10).

Additionally, the independent validation group dataset includes smoking habit information, allowing us to further investigate its impact on BA predictions by comparing performance across smokers and nonsmokers. By examining the angular difference (φ) among individuals, we find that the smoking subgroup of females shows no significant difference in φ compared to their nonsmoking counterparts (Fig. 4B, Female). In contrast, the smoking subgroup of males exhibits a statistically significant difference in φ when compared to nonsmokers (Fig. 4B, Male), suggesting that smoking habits may accelerate biological aging in male individuals (*49*, *50*).

It is important to note that while the modeling group lacks explicit smoking habit labels, which obscures the absolute positioning of the reference frame, the relative distribution of φ among different smoking habits in the validation group remains discernible. Specifically, when using the model group data as a baseline, the test of φ against a value of zero shows no significant differences across both female and male model groups. However, the relative distribution differences in φ among the validation group’s smoking habits are preserved, highlighting the robustness of our DNN model in capturing the nuanced effects of lifestyle factors on biological aging.

### Sensitivity analysis for identifying aging key markers

**Fig. 5.**
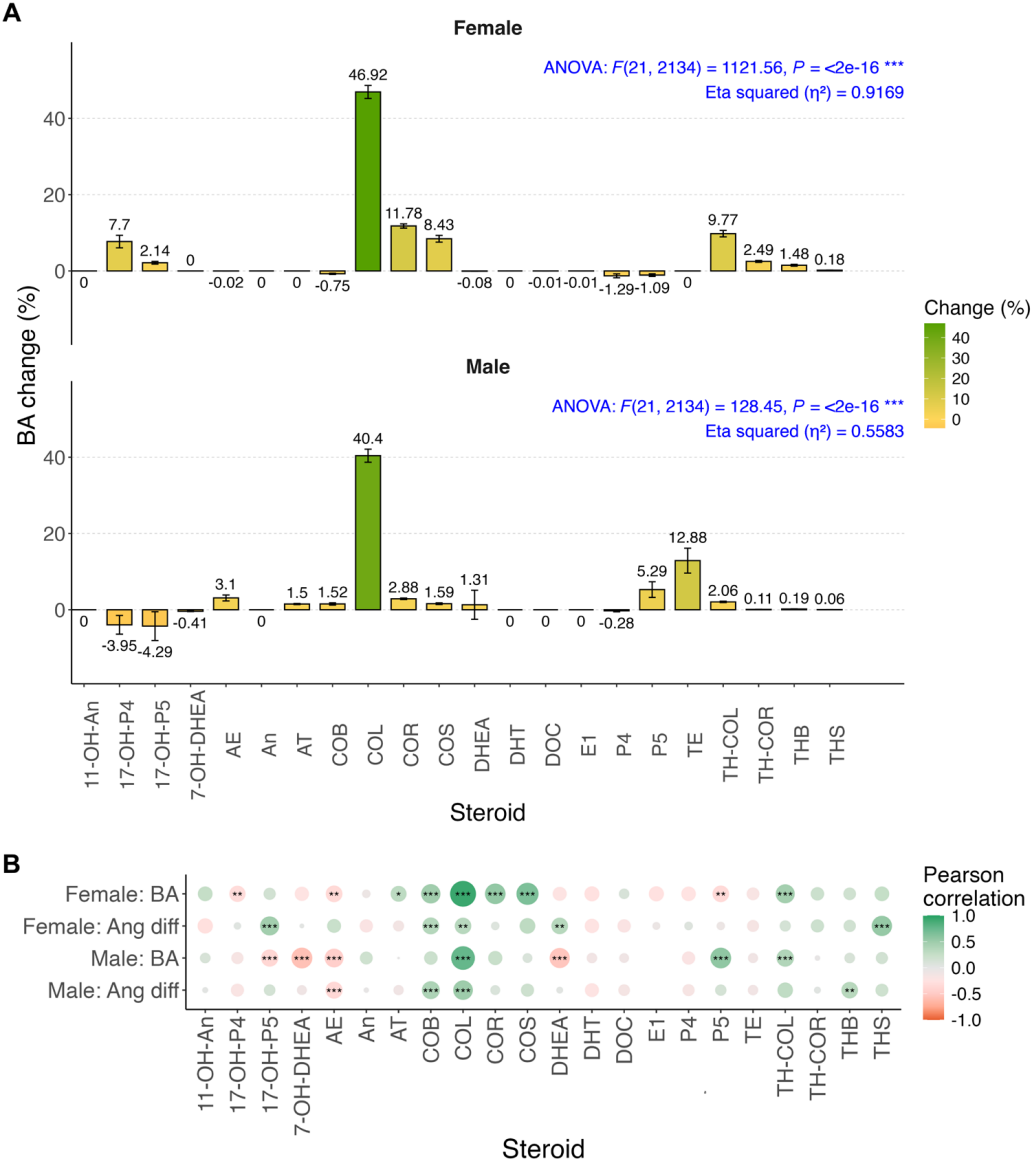
Sensitivity analysis and aging marker identification. (**A**) Percentage change in BA in response to a twofold increase in each of the 22 steroid input nodes for sex-specific DNN models. Error bars represent the variability in sensitivity results across individuals. (**B**) Heatmaps of Pearson correlation coefficients between the 22 steroid input nodes and BA, as well as the angular difference between BA and CA in both female and male models. Pearson correlation test was assessed with Bonferroni correction. ns *P*_adj_ ≥ 0.05; *P_adj_ < 0.05; **P_adj_ < 0.01; ***P_adj_ < 0.001.

To assess the sensitivity of the established DNN model, we examined the impact of doubling the input values of each steroid node on the output values of the BA node (Fig. 5A and table S8). Notably, both the female and male DNN models revealed that cortisol (COL), a steroid associated with stress and present in relatively high concentrations, exerted a momentous positive sensitivity effect on BA predictions, exceeding 40%. Additionally, in the female model, steroids such as 17α-hydroxyprogesterone (17-OH-P4), cortisone (COR), 11-deoxycortisol (COS), and TH-COL also demonstrated a stable positive influence on BA. In the male model, P5 and testosterone (TE) exhibited similar trends.

Analysis of variance (ANOVA) results indicated that the input values of the 22 steroids explained a substantial portion of the BA prediction model, achieving an explanatory ability (η^2^ value) of 0.9169 for females and 0.5583 for males, highlighting the reliability of our biological process modeling. Conversely, we did not identify any steroids with a consistent negative impact on BA, suggesting that the physiological regulation required to delay biological aging is more likely associated with the reduction of stress-related hormones.

Furthermore, an analysis of the scaled values of each steroid concerning smoking habits revealed no significant differences across sexes (fig. S11), underscoring the necessity of considering the biological process among steroids. When evaluating BA as an absolute indicator of aging, alongside the angular difference (φ) of BA against CA as a relative aging indicator, COL demonstrated a strong linear correlation and high confidence for both indicators across sex models (Fig. 5B). This suggests that COL may serve as a robust marker for reflecting BA, with its associated physiological pathways likely containing important factors or processes related to aging regulation.

## DISCUSSION

Here, we developed a DNN model based on steroid metabolic pathways to predict BA. The model effectively captured the increasing heterogeneity of aging over time and the complex biological processes influenced by steroidogenesis. After training the model on a well-organized dataset, we analyzed the intricate relationships between specific hormones and physiological aging processes. The DNN architecture allowed for a detailed examination of how different steroids affect BA, revealing notable sex-specific differences between female and male models. These findings underscore the distinct metabolic pathways in each sex and their influence on aging trajectories.

Steroid metabolism is a systemic process involving contributions from multiple organs, including the adrenal glands, liver, and gonads (*51*). The resulting steroid profiles provide an integrated perspective on biological aging, capturing dynamic changes that transcend isolated organ-specific dysfunction. By focusing on steroid metabolic pathways, our approach bridges the gap between organ-specific changes and systemic aging patterns, offering a foundation for investigating the mechanisms underlying aging heterogeneity. This systemic nature complements the use of CA as an objective temporal reference during model training, allowing us to quantify deviations arising from physiological and metabolic heterogeneity and to contextualize relative biological changes within an absolute chronological framework. These findings highlight the utility of steroid profiles as dynamic biomarkers for investigating the multifaceted nature of aging.

One strength of our model lies in its use of a CDF-based proportional scaling that preserves intrinsic relative proportions of steroid concentrations while minimizing variations stemming from experimental batch effects, individual physiological differences, and potential circadian fluctuations (*52*, *53*). Building on the observed interindividual synchronicity in steroid profiles, this approach effectively accounts for subtle variability while maintaining synchronization across individuals. As a result, it greatly enhances the robustness and accuracy of predictions, as validated within our independent datasets. However, broader applicability to external datasets remains constrained by the requirement for a full steroid panel. Future refinements could explore alternative normalization strategies, such as leveraging total cholesterol as a reference, to facilitate model adaptation to datasets with fewer available steroid measurements while preserving predictive accuracy.

Our analysis revealed several steroid markers associated with aging, with COL standing out as a key factor. While these markers have been previously identified, our unique analytical framework offers deeper insights by providing a refined perspective on their biological relevance. Specifically, we observed a positive correlation between COL and BA, supporting its role as a stress biomarker that reflects cumulative physiological wear and tear. This finding aligns with studies examining the complex relationship between stress, aging, and steroid metabolism. While COL may act as a marker for aging rather than a direct causative agent, its involvement in processes such as gluconeogenesis and inflammation suppression calls for further investigation into its upstream and downstream pathways.

When considering lifestyle factors, smoking status emerged as a notable variable, although our validation cohort lacked detailed information on other behaviors such as alcohol consumption and diet (*54*–*59*). Our results revealed that only male smokers exhibited a more accelerated aging trajectory compared to nonsmokers. We hypothesize that this disparity may be due to the generally higher smoking frequency among males. In contrast, the lower smoking frequency in females, combined with unmeasured lifestyle factors that influence BA, may have obscured the aging effects in female smokers. Future studies with larger cohorts and more comprehensive lifestyle and physiological data will be crucial to further elucidate these relationships.

Despite the valuable insights provided by this study, several limitations should be acknowledged. The relatively small sample size and the lack of detailed lifestyle data across certain cohorts limit the generalizability of our findings. Additionally, our treatment of steroids in a static manner, without fully accounting for their broader biochemical pathways and circadian variations, may introduce biases. Furthermore, the specific design of our DNN model, tailored to reflect the complexity of steroidogenesis pathways and aging heterogeneity, presents challenges for direct comparisons with traditional methods (*17*, *40*, *41*) or other neural network designs (*60*). These challenges arise from fundamentally different evaluation criteria and objectives—balancing biological interpretability with predictive accuracy in our framework.

To address these limitations, future research should focus on longitudinal studies that track individuals over time and adopt more dynamic approaches that capture steroid fluctuations. Expanding the dataset to encompass a broader range of environmental and behavioral factors, alongside deeper investigations of sex-specific metabolic pathways, will further enhance the predictive power and clinical relevance of the model. Additionally, comparative analyses with alternative modeling approaches on larger and more diverse datasets could provide further insights into the strengths and weaknesses of our approach, enabling a more comprehensive evaluation of its utility. These advancements could help establish the BA prediction model a valuable tool in personalized aging interventions, facilitating the identification of biomarkers and enabling more targeted strategies to modulate aging processes.

## MATERIALS AND METHODS

### Chemicals

This study used 30 steroid standards and 14 internal standards, as summarized in fig. S1 and table S1. HPLC-grade methanol (MeOH), acetonitrile (ACN), and 99.998% trace metals basis lithium chloride (LiCl), as well as analytical reagent–grade acetic acid (AcOH), were purchased from Sigma-Aldrich (USA). HPLC-grade formic acid was obtained from FUJIFILM Wako Pure Chemical Corporation (Japan). Ultrapure water was produced using an Organo Puric ω system (Japan).

### Sample acquisition and cohort

Serum samples from 150 healthy individuals were obtained from BIOIVT (New York, US). All samples were collected in the United States, imported to Japan on dry ice, and stored at −80°C until analysis. The storage and study protocols were approved by the Institute for Protein Research’s ethics committee. Our study modeled BA using data from 100 healthy participants (50 females and 50 males, aged 20 to 73 years). The model was then applied to a validation cohort of 50 participants (25 females and 25 males, aged 40 to 59 years). Of the validation set, 40 participants were nonsmokers, while 10 were smokers, each smoking at least 10 cigarettes per day.

### Sample preparation

Serum (240 μl) and 4.8 μl of isotope-labeled internal standard solution were added to a 1.5-ml tube (Eppendorf, Germany) and mixed, followed by the addition of 480 μl of ACN. The mixture was vortexed at 3200 rpm for 30 s and incubated at 4°C for 30 min, followed by centrifugation at 20,000*g* at 4°C for 15 min to precipitate proteins. The supernatant was transferred to a 15-ml tube and diluted with 4.08 ml of H_2_O, achieving a final ACN concentration of 10% (v/v). After a second centrifugation at 19,000*g* at 4°C for 15 min, the supernatant was applied to a Bond Elut C18 column (Agilent, USA), preconditioned with 80% ACN and 10% ACN. The sample was washed with 10% ACN and eluted with 1 ml of 80% ACN, which was collected in a clean tube. The eluate was dried using a speed-vac, and the residue was redissolved in 24 μl of 40% MeOH. After centrifugation at 20,000*g* for 15 min, the supernatant was transferred and analyzed by LC-MS/MS.

### LC-MS/MS analysis

LC-MS/MS quantification of steroids was performed using an Agilent 1290 Infinity II and a 6470 triple quadrupole mass spectrometer (Agilent) in positive ion mode, using the multiple reaction monitoring (MRM) method. Chromatographic separation was achieved with a C18 column (Eclipse Plus C18 RRHD 2.1 × 100 mm, 1.8 μm, Agilent, USA) at 40°C. The details of this method are reported in our previous method.

### R environment and packages

All computational analyses were performed in R (version 4.4.1). A complete list of packages and their versions used in this study is provided in table S9. These packages were used for data preprocessing, statistical analysis, and model construction.

### CDF-based proportional scaling

To achieve cross-batch alignment while maintaining within-group proportionality, we calculated a scaling factor *k* for each individual dataset. This process began by applying the Yeo-Johnson transformation (*61*) $X\prime =f_{\text{YJ}}\left(X,\lambda \right)$ to each variable **X**(representing the original concentration of steroids) to address nonnormality. Here, X′ represents the transformed data and $f_{\text{YJ}}$ is the transformation function

$$f_{\text{YJ}}\left(x;\lambda \right)=\left\{ \begin{array}{cc} \frac{{\left(x+1\right)}^{\lambda }-1}{\lambda }\; & \text{if}\;\lambda \neq 0,x\geq 0\\ \text{log}\left(x+1\right)\; & \text{if}\;\lambda =0,x\geq 0\\ -\frac{{\left(-x+1\right)}^{2-\lambda }-1}{2-\lambda }\; & \text{if}\;\lambda \neq 2,x<0\\ -\text{log}\left(-x+1\right)\; & \text{if}\;\lambda =2,x<0 \end{array}\right.$$ (1)

Here, λ was optimized for each variable (steroid) within the modeling group and retained as a parameter for transformations on independent validation data. Next, to standardize across individuals, we computed the *z* score as

$$Z=\frac{\left(X\prime -\mu \right)}{\sigma }$$ (2)

where μ is the mean and σ is the SD of X′ within each steroid, both optimized within the modeling group and retained for parameterized normalization on independent validation data. Thus, for independent validation data, transformations and normalizations in Eqs. 1 and 2 were performed using the retained parameters λ, μ, and σ from the modeling group. The *z* scores **Z** were then mapped to a common CDF to derive the scaling factor *k*

$$CDF\left(x;\mu ,\sigma \right)=\frac{1}{\sigma \sqrt{2\pi }}\int_{-\infty }^{x}\text{exp}\left[-\frac{{\left(t-\mu \right)}^{2}}{2\sigma^{2}}\right]\mathrm{dt}$$ (3)

$$k\equiv \frac{0.5}{\mu_{CDF}}$$ (4)

In Eq. 3, μ is the mean of the distribution and σ is its SD. In Eq. 4, $\mu_{CDF}$ is the mean CDF value across all steroids for each individual, with 0.5 representing the cumulative distribution at *Z* = 0. Finally, each individual’s primary-derived dataset was rescaled by *k* to achieve aligned concentration values

$$X_{\text{scaled}}=k\cdot X$$ (5)

Here, $X_{\text{scaled}}$ denotes the final scaled concentration, preserving proportional consistency across batches.

### Metabolic pathway–based DNN architecture

We extracted steroid-associated metabolic pathways from the Kyoto Encyclopedia of Genes and Genomes (KEGG) database (*62*, *63*) and encoded each pathway as input to a deep multilayer perceptron (DNN), designed to simulate steroid synthesis and metabolic processes in organisms. The DNN model was built with 25 components, beginning with P5, continuing through 21 intermediate steroids, and concluding with PI and SI outputs, with a final output for BA. To enhance biological interpretability, the model incorporated 22 input nodes representing scaled data for P5 and the 21 intermediate steroids, distributed across eight layers. It also included 24 bias nodes (including PI, SI, and BA) to capture influences from other biological processes. These input and bias nodes collectively fed into 24 join nodes, which represent intermediate outputs for the 21 steroids, PI, and SI, as well as the final BA output. A total of 37 weighted edges linked upstream input or join nodes to downstream ones, capturing interactions among these biological components.

### Model loss function designing

WSATL function was designed to address the expanding heterogeneity of aging over time

$$L\left(y,\hat{y}\right)\equiv \frac{{\left\{\sqrt{{\left[arctan\left(\frac{y}{\hat{y}}\right)-arctan\left(\frac{\hat{y}}{y}\right)\right]}^{2}+\delta^{2}}-\delta \right\}}^{2}}{1+\sqrt{{\left(y-\hat{y}\right)}^{2}+\delta^{2}}-\delta }$$ (6)

where *y* represents the true CA and $\hat{y}$ represents the predicted BA. The parameter δ (default set to 0.001) smooths the function $f\left(x\right)=\sqrt{x^{2}+\delta^{2}}-\delta$, mitigating sharp deviations. The angular difference between $arctan\left(\frac{y}{\hat{y}}\right)$ and $arctan\left(\frac{\hat{y}}{y}\right)$ captures proportional similarity between BA and CA, while the denominator in Eq. 6 acts as a weighting factor, imposing greater penalties as predictions near true values to avoid overfitting.

### Model training strategy

Weighted edges between steroids were initialized based on their Spearman correlation coefficients, with edges from steroids to PI or SI set according to their correlations with CA. Edges from PI and SI to BA were intuitively set to 1 or −1, based on previous findings associating stress with aging acceleration and active sex hormone levels with youthfulness. Join nodes for intermediate steroids were processed through the activation function ReLU(x)=max(0,x), while other join nodes remained linear. The weights of all edges and biases were iteratively updated through backpropagation, managed by the Adam optimizer (*64*) with default momentum parameters β_1_ = 0.9, β_2_ = 0.999, and ϵ = 10^−8^.

### Hyperparameter tuning via cross-validation

Hyperparameters in DNN training, specifically the learning rate (*lr*) and epochs (*t*), were optimized using fivefold cross-validation. The modeling group data were divided into five subsets, with each subset serving iteratively as the validation-fold while the remaining four formed the training-fold data. Optimal values for *lr* and *t* were determined by balancing four criteria: median validation loss, the difference in median loss between validation and training folds, SD differences in loss between folds, and the training efficiency ratio (*t*/*lr*). This approach ensured robust model performance across a range of learning rates (*lr*: 0.001, 0.003, 0.005, 0.01) and epochs (*t*: 1000, 2000, 3000, 4000, 5000, 6000, 7000, 8000, 9000, 10,000).

### Performance validation across sex and lifestyle factors

After training, the DNN model was transferred to an independent validation dataset to assess its performance. Loss values served as metrics to determine whether there were statistically significant performance differences between the training and validation datasets across sexes, using the Wilcoxon rank sum test with Bonferroni correction and Kruskal-Wallis *H* test.

The model’s performance was further evaluated using the defined angular difference φ, calculated as

$$\varphi \equiv arctan\left(\frac{\hat{y}}{y}\right)-\frac{\pi }{4}$$ (7)

where *y* represents the true CA and $\hat{y}$ represents the predicted BA. We tested whether the median φ values for each group (modeling, smoking, and nonsmoking) significantly differed from zero (H_0_: median = 0) using the Wilcoxon test with Bonferroni correction. Additionally, to examine the effects of smoking status, we assessed sex-specific differences in φ values using the Wilcoxon test, with Bonferroni correction applied. This analysis aimed to reveal how smoking habits may affect the model’s predictive capacity, providing insights into the potential impact of lifestyle factors on BA predictions.

### Sensitivity analysis of the DNN model

To evaluate the sensitivity of the DNN model to variations in steroid levels, each steroid concentration was independently increased by 100% within the dataset. For each steroid, the resulting change in predicted BA was calculated as a percentage difference from the baseline prediction. These calculations were performed across the entire modeling group, stratified by sex. Using these individual BA changes, we derived the mean change and 95% confidence intervals (CIs), expressed as CI = μ_Change_ ± 1.96 × σ_Change_, to quantify each steroid’s impact on model predictions. An ANOVA test on the BA changes assessed significant sensitivity differences across steroids, followed by an effect size (η^2^) calculation. Post hoc Tukey’s Honestly Significant Difference (HSD) analysis further identified steroids with distinct sensitivity effects.
