# Supplementary material for: Biological age prediction using a DNN model based on pathways of steroidogenesis
Source: Sci Adv. 2025 Mar 14;11(11):eadt2624. doi: 10.1126/sciadv.adt2624 (PMC11908500; doi:10.1126/sciadv.adt2624)
Supplement: Supplementary file 1 — Supplementary Text Figs. S1 to S11 Legends for tables S1 to S9 [file sciadv.adt2624_sm.pdf]

Science Advances
AAAS

Supplementary Materials for

Biological age prediction using a DNN model based on pathways of steroidogenesis

Qiuyi Wang *et al.*

Corresponding author: Zi Wang, wang.zi@protein.osaka-u.ac.jp; Toshifumi Takao, tak@protein.osaka-u.ac.jp

Sci. Adv. **11**, eadt2624 (2025)
DOI: 10.1126/sciadv.adt2624

The PDF file includes:

Supplementary Text
Figs. S1 to S11
Legends for tables S1 to S9

Other Supplementary Material for this manuscript includes the following:

Tables S1 to S9

Supplementary Text

Preparation of standard stock, calibration, and quality control stock solutions

Each steroid standard was prepared in methanol at a concentration of 4 mg mL^{-1} as respective stock solutions and stored in -80 °C. These stock solutions were mixed and diluted to 10 ng μL^{-1} and with 40% MeOH as mixed stock solutions at -80 °C. The working standard solutions were prepared at concentrations of 0.4, 1, 2, 4, 10, 20, 40, 100, 160, 200, 400, 600, 1000, 1600, 2000, 4000 pg μL^{-1} with 40% MeOH for 16-OH-E1,7-OH-DHEA, TH-COL, 7-OH-P5, TH-COR, 11-OH-An, THB, APD, 17-OH-P5, 3β,5αTH-DOC, THS, 3α,5β-TH-DOC, COR, AT, COL, COB, COS, AE, E1, E2, DOC, TE, 17-OH-P4, DHT, and P4, and at concentrations of 0.8, 2, 4, 8, 20, 40, 80, 200, 320, 400, 800, 1200, 2000, 3200, 4000, 8000 pg μL^{-1} with 40% MeOH for DHEA, P5, E3, An and al-P5. An internal standard (IS) mixture solution of 0.1 ng μL^{-1} of 17-OH-P4-$^{13}C_3$, TE-$^{13}C_3$, AE-$^{13}C_3$, COR-$^{13}C_3$, P4-$^{13}C_3$, TH-COR-d_6 and 16-OH-E1-$^{13}C_3$, and 0.2 ng μL^{-1} of COL-d_4, COB-d_4, E1-$^{13}C_3$, E3-d_3 and 17-OH-P5-d_3, and 0.4 ng μL^{-1} of DHEA-d_5 and P5-$^{13}C_2d_2$ was prepared in 40% MeOH.

For calibration and quality control samples, 24 μL of blank matrix (see below) was spiked with 6 μL of working standard solution and 4.8 μL of IS solution. The samples were evaporated to dryness and re-dissolved in 24 μL of 40% MeOH. Finally, the calibration samples were at levels of 0.01, 0.02, 0.05, 0.1, 0.2, 0.5, 1, 2, 5, 8, 10, 20, 30, 50, 80, 100, 200 pg μL^{-1} for 16-OH-E1,7-OH-DHEA, TH-COL, 7-OH-P5, TH-COR, 11-OH-An, THB, APD, 17-OH-P5, 3β,5αTH-DOC, THS, 3α,5β-TH-DOC, COR, AT, COL, COB, COS, AE, E1, E2, DOC, TE, 17-OH-P4, DHT, and P4, and at levels of 0.02, 0.04, 0.1, 0.2, 0.4, 1, 2, 4, 10, 16, 20, 30, 50, 100, 160, 200, 400 pg μL^{-1} for DHEA, P5, E3, An and al-P5.

Preparation of blood sample blank matrix for LC-MS/MS validation

For method validation, charcoal was added during the sample preparation procedure. Serum samples were protein precipitated with ACN. After centrifuging, the supernatant was collected and diluted with H_2O until 10% ACN. Then charcoal was added to strip the steroids from the system. 0.6 mg of activated charcoal was added per microliter of serum, followed by vortexing and centrifugation. The supernatant was loaded on the bond elute column for further purification. The eluate was evaporated to dryness with speed-vac and re-dissolved with 40% MeOH to form 10 $\mu L \cdot \mu L^{-1}$ blank matrix.

Since the concentration of sex steroid hormones are extremely low in the serum of very old people, researchers used such serum as sex steroid free blank matrix. However, the corticosteroids play important roles human body regardless of age, it was impossible to find the serum samples for which the corticosteroid did not exist. In this case, charcoal extraction was used for the blank matrix preparation procedure. In should be noted that charcoal might strip out the interfering compounds, which might be the weakness of using a blank matrix prepared in this way.

LC-MS/MS method validation

The developed method was satisfactorily validated in terms of the LOQ, linear range, extraction recovery, precision and accuracy.

Calibration curves and LOQ: The calibration curves, correlation coefficients, linear ranges, and LOQs of the 13 steroids in the spiked blank matrix are shown in Table S3. The calibration

curve was constructed using the peak area ratios of a compound to IS versus the ratios of concentrations of a compound at different levels to the concentration of IS. The correlation coefficient square (r^2) was calculated. LOQ was tested at a signal to noise (S/N) of 10. Good linearity was observed for all 29 steroids within the ranges (0.01-10 pg μL^{-1} for 7-OH-DHEA, 0.02-2 pg μL^{-1} for 3β,5α-TH-DOC, 0.02-5 pg μL^{-1} for 16-OH-E1, 0.02-20 pg μL^{-1} for TH-COR, 0.04-10 pg μL^{-1} for DOC, 0.05-10 pg μL^{-1} for AT, 0.05-20 pg μL^{-1} for COS, 0.05-50 pg μL^{-1} for 7-OH-P5, and TH-COL, 0.1-8 pg μL^{-1} for THB, 0.1-20 pg μL^{-1} for 17-OH-P4 and P4, 0.1-50 pg μL^{-1} for 11-OH-An and 17-OH-P5, 0.2-20 pg μL^{-1} for APD, THS, 3α,5β-TH-DOC, COB, AE, E1, E2, TE, DHT and An, 0.4-100 pg μL^{-1} for P5, 0.5-50 pg μL^{-1} for COR, 1-100 pg μL^{-1} for DHEA, E3 and al-P5, and 2-200 pg μL^{-1} for COL. The linear correlation coefficient square (r^2) was greater than 0.9921. The lower LOQ was 0.005-0.288 pg μL^{-1} for serum, suggesting that the developed method is highly sensitive for the quantification of the steroids.

Matrix effect and recovery in LC-MS/MS analysis: To evaluate the matrix effect (ME) and recovery (R), low (0.05, 0.2, 0.4, 1, 2, 5, 10 or 50 pg μL^{-1}), medium (0.2, 0.4, 1, 2, 5, 10, 20 or 80 pg μL^{-1}) and high (1, 2, 5, 10, 20, 40 or 100 pg μL^{-1}) concentrations of spiked blank matrix and serum samples (n = 3) were assessed. The matrix effect value was calculated as ME (%) = $A_{matrix}/A_{solution} \times 100$, where $A_{solution}$ is the compound peak area of 10 µL of pure standard and A_{matrix} is the compound peak area of blank matrix spiked with 10 µL standard sample. The recovery value was calculated as R (%) = $A_{pre\text{-}spike}/A_{post\text{-}spike} \times 100$, where $A_{pre\text{-}spike}$ is the compound peak area of serum spiked with 10 µL standards before extraction and $A_{post\text{-}spike}$ is the compound peak area of serum spiked with 10 µL standards after extraction. The extraction matrix effect and recoveries (%) were within the range of 88.09-127.64.40% and 75.15-121.00% in Table S3.

Accuracy and precision in LC-MS/MS analysis: The precision and accuracy of the method were assessed by performing six replicates of serum samples spiked with low (0.05, 0.2, 0.4, 1, 2, 5, 10 or 50 pg μL^{-1}), medium (0.2, 0.4, 1, 2, 5, 10, 20 or 80 pg μL^{-1}) and high (1, 2, 5, 10, 20, 40 or 100 pg μL^{-1}) of steroids (Table S3). Accuracy was calculated as the averaged percentage for the measured concentrations to the real concentrations. Precision was expressed as the RSDs (relative standard deviation) of the measured concentrations and was performed on three separate days. The low and high concentration of steroids spiked in the intra- and inter-batch samples were obtained within acceptable ranges. Table S3 summarizes the accuracy and precision data for serum. The results indicated that the method shows a moderately good precision and accuracy. The accuracy (%) was 74.75-113.05 while the intra-day precision (%) was 0.002-17.65 and the Inter-day precision (%) was 0.001-23.85. The method was considered to be suitable in terms of accuracy and precision.

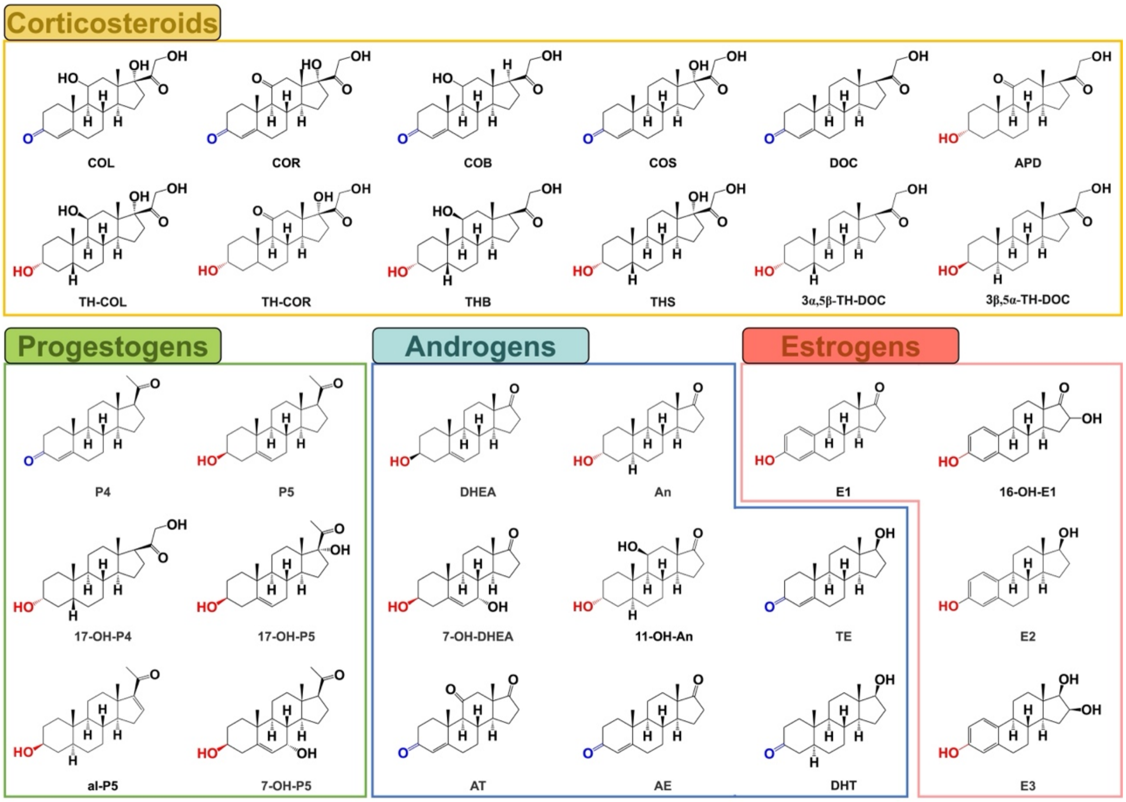

Fig. S1. Structures of steroids analyzed in this study.

The steroid structures are categorized by color-coded boxes: yellow for corticosteroids, pink for estrogens, green for progestogens, and blue for androgens. 11-OH-An, 11-β-hydroxyandrosterone; 16-OH-E1, 16-hydroxyestrone; 17-OH-P5, 17α-hydroxypregnenolone; 17-OH-P4, 17α-Hydroxyprogesterone; 7-OH-DHEA, 7α-hydroxydehydroepiandrosterone; 7-OH-P5, 7α-hydroxypregnenolone; Androstenedione, AE; al-P5, allopregnenolone; An, androsterone; APD, alphadolone; AT, Adrenosterone; COB, Corticosterone; COL, Cortisol; COR, cortisone; COS, 11-Deoxycortisol; DHEA, dehydroepiandrosterone; DHT, dihydrotestosterone; DOC, 11-Deoxycorticosterone; E1, Estrone; E2, Estradiol; E3, estriol; P4, Progesterone; P5, pregnenolone; TE, Testosterone; THB, tetrahydrocorticosterone; TH-COL, tetrahydrocortisol; TH-COR, tetrahydrocortisone; TH-DOC, 3β,5α-tetrahydrodeoxycorticosterone; THS, tetrahydrodeoxycortisol.

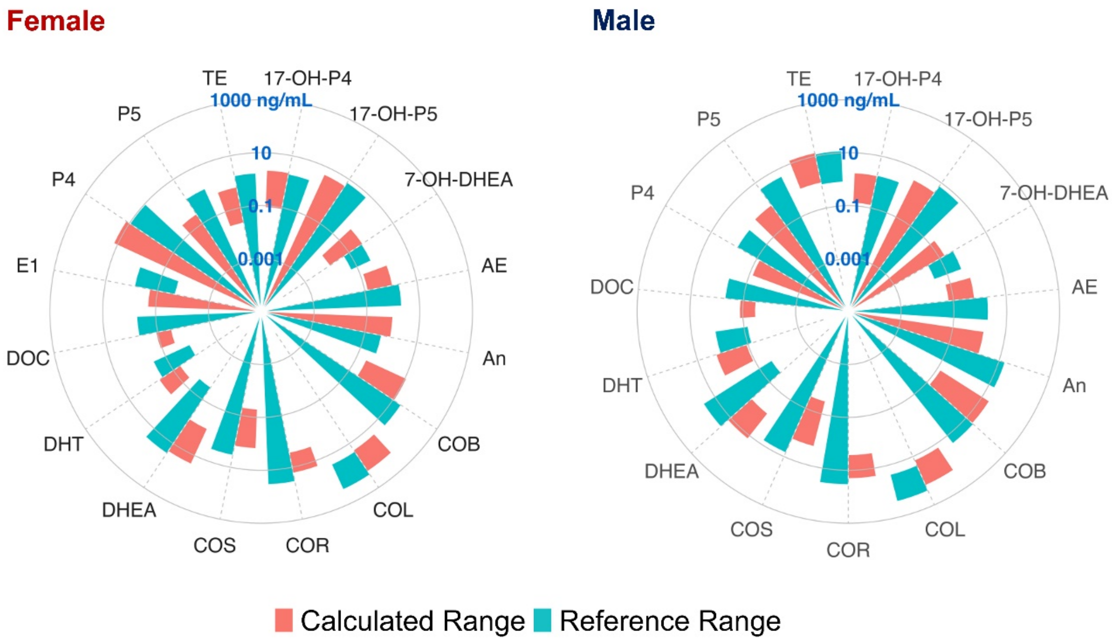

Fig. S2. Comparison of steroid concentrations with published reference ranges.

Radar plots compare steroid concentrations measured by the developed method (red bars) with reference ranges from the literature (blue bars) for both male and female healthy subjects. Detailed reference ranges are summarized in Table S5.

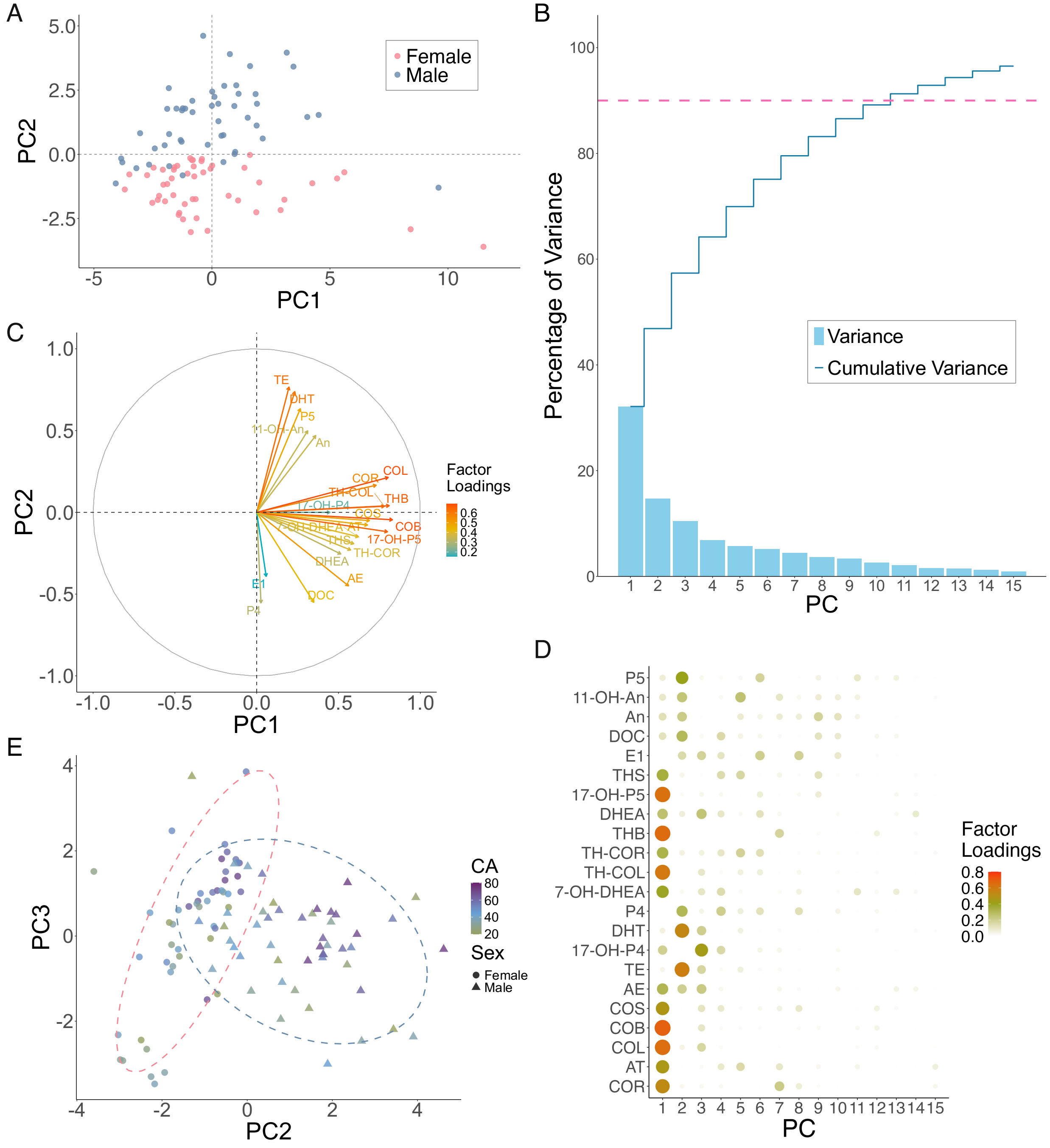

Fig. S3. Principal component analysis (PCA) of the original modeling dataset.

PCA results of the original modeling dataset with one replicate (female, n = 49; male, n = 49) include (**A**) a scatter plot of PC1 vs. PC2, illustrating the relationship between sex and principal components (PCs); (**B**) a bar plot showing the percentage of variance explained and cumulative variance for each PC, with a dashed red line indicating 90% of the explained variance; (**C**) factor loadings of each steroid on PC1 and PC2, highlighting their contributions to these two principal components; (**D**) factor loadings of each steroid across multiple PCs, providing an overview of how steroid variables are distributed across different dimensions; and (**E**) a scatter plot of PC2

vs. PC3, highlighting the relationship between chronological age (CA) and PCs, with dashed ellipses representing the 95% coverage of the sample distribution for each sex, calculated based on a multivariate t-distribution (pink for females, navy for males).

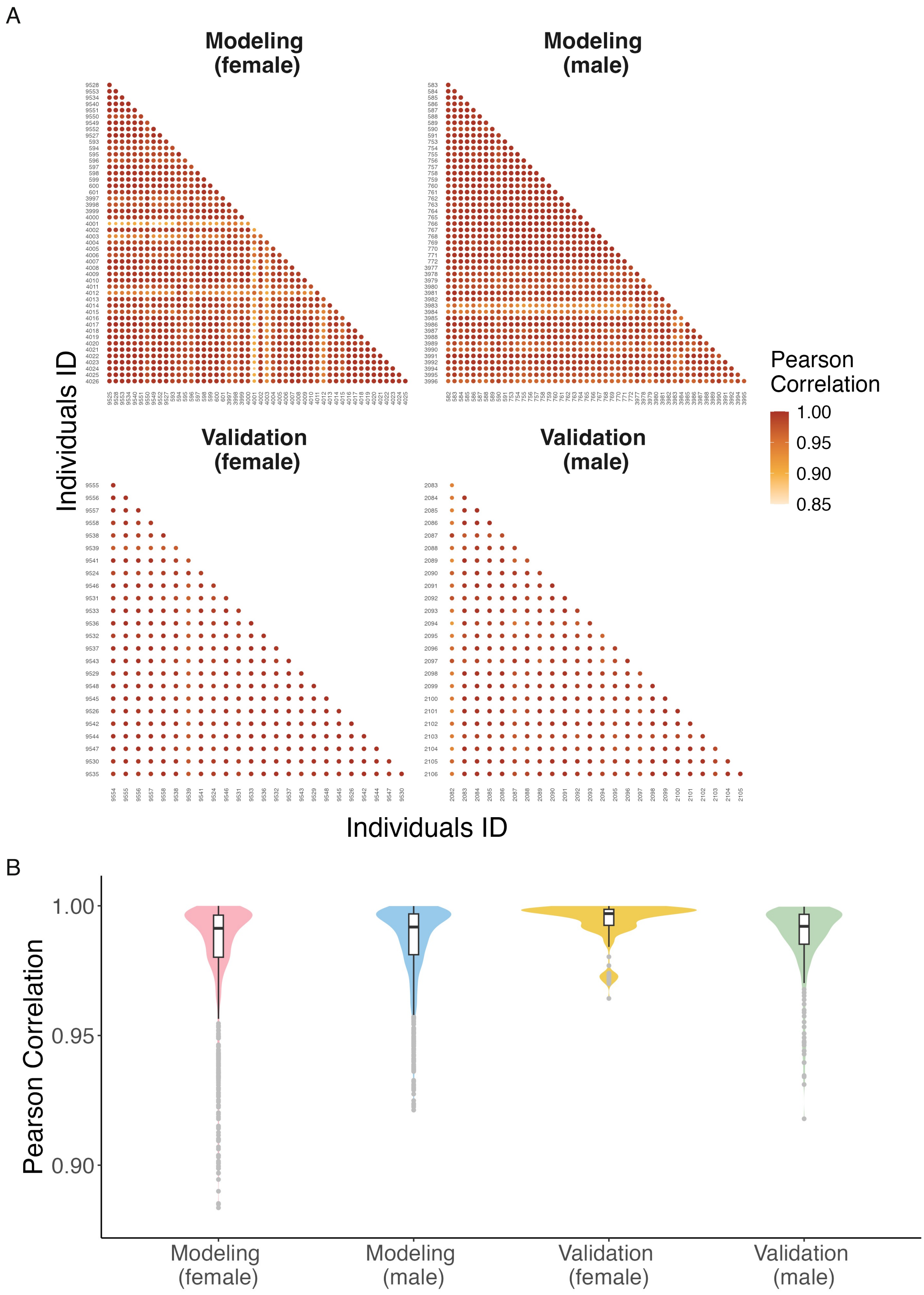
A
Modeling (female)
Modeling (male)
Validation (female)
Validation (male)
Individuals ID
Individuals ID
Pearson Correlation
1.00
0.95
0.90
0.85
B
Pearson Correlation
1.00
0.95
0.90
Modeling (female)
Modeling (male)
Validation (female)
Validation (male)

Fig. S4. Pearson correlation coefficients across individuals.

The correlation analysis results include (**A**) a heatmap of Pearson correlation coefficients between individuals across all groups with one replicate: modeling dataset (female, n = 49; male, n = 49) and independent validation dataset (female, n = 25; male, n = 25); and (**B**) a violin plot overlaid with boxplot displaying the distribution of Pearson correlation coefficients within each dataset.

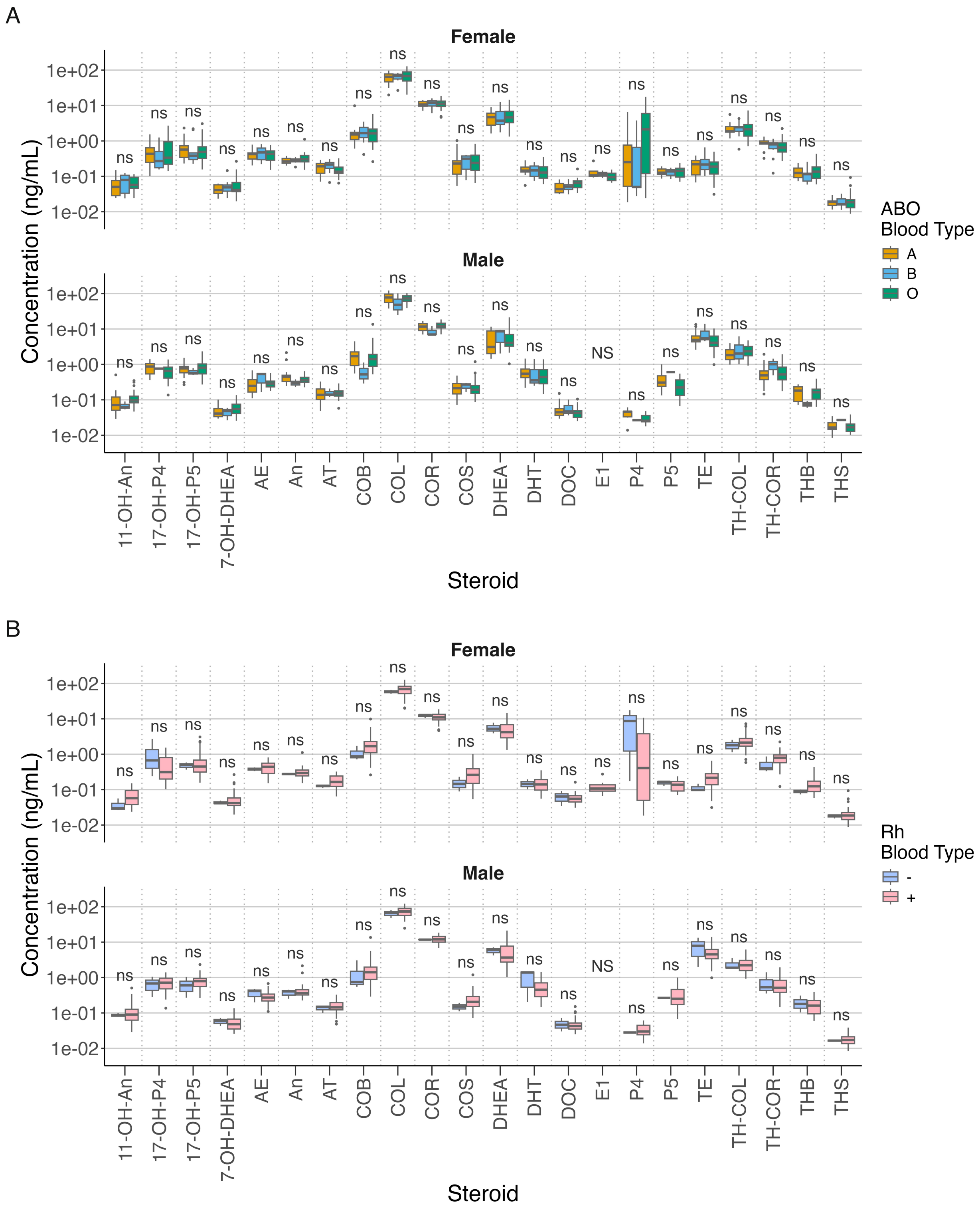

Fig. S5. Distribution of steroid concentrations before and after scaling for different blood types.

Univariate analysis for (**A**) ABO blood type and (**B**) Rh blood type was performed using the Kruskal-Wallis test with Bonferroni correction. The data for ABO blood type include females (A: n = 11; B: n = 11; O: n = 26; AB: n = 1, excluded from analysis) and males (A: n = 16; B: n = 3; O: n = 30) from the modeling dataset. The Rh blood type data include females (+: n = 46; -: n = 3) and males (+: n = 46; -: n = 3) also derived from the modeling dataset. Statistical significance, NS, all zero values (non-test); ns, $P_{adj} \geq 0.05$.

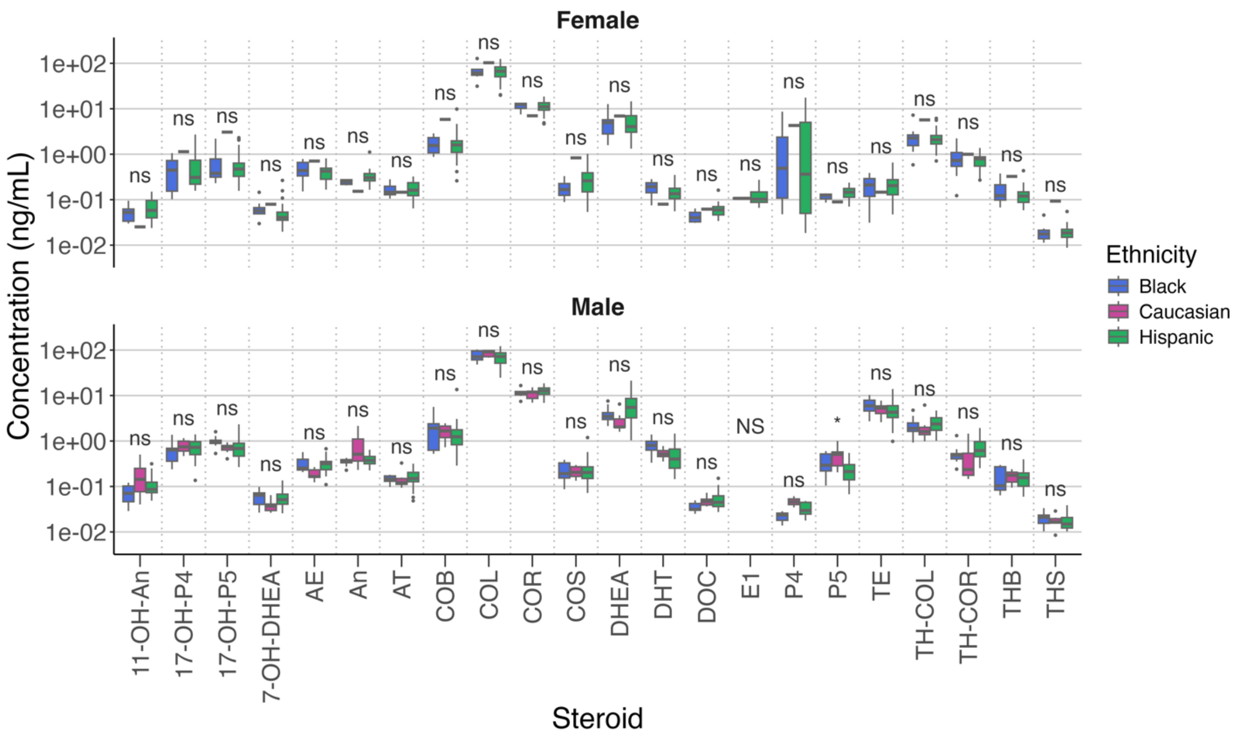

Fig. S6. Distribution of steroid concentrations before and after scaling for different ethnicities.

Univariate analysis for ethnicity, using the Kruskal-Wallis test adjusted by Bonferroni correction. The data for ethnicity include females (Black: n = 8; Caucasian: n = 1; Hispanic: n = 40) and males (Black: n = 9; Caucasian: n = 7; Hispanic: n = 33) from the modeling dataset. Statistical significance, NS, all zero values (non-test); ns, $P_{adj} \geq 0.05$; * $P_{adj} < 0.05$.

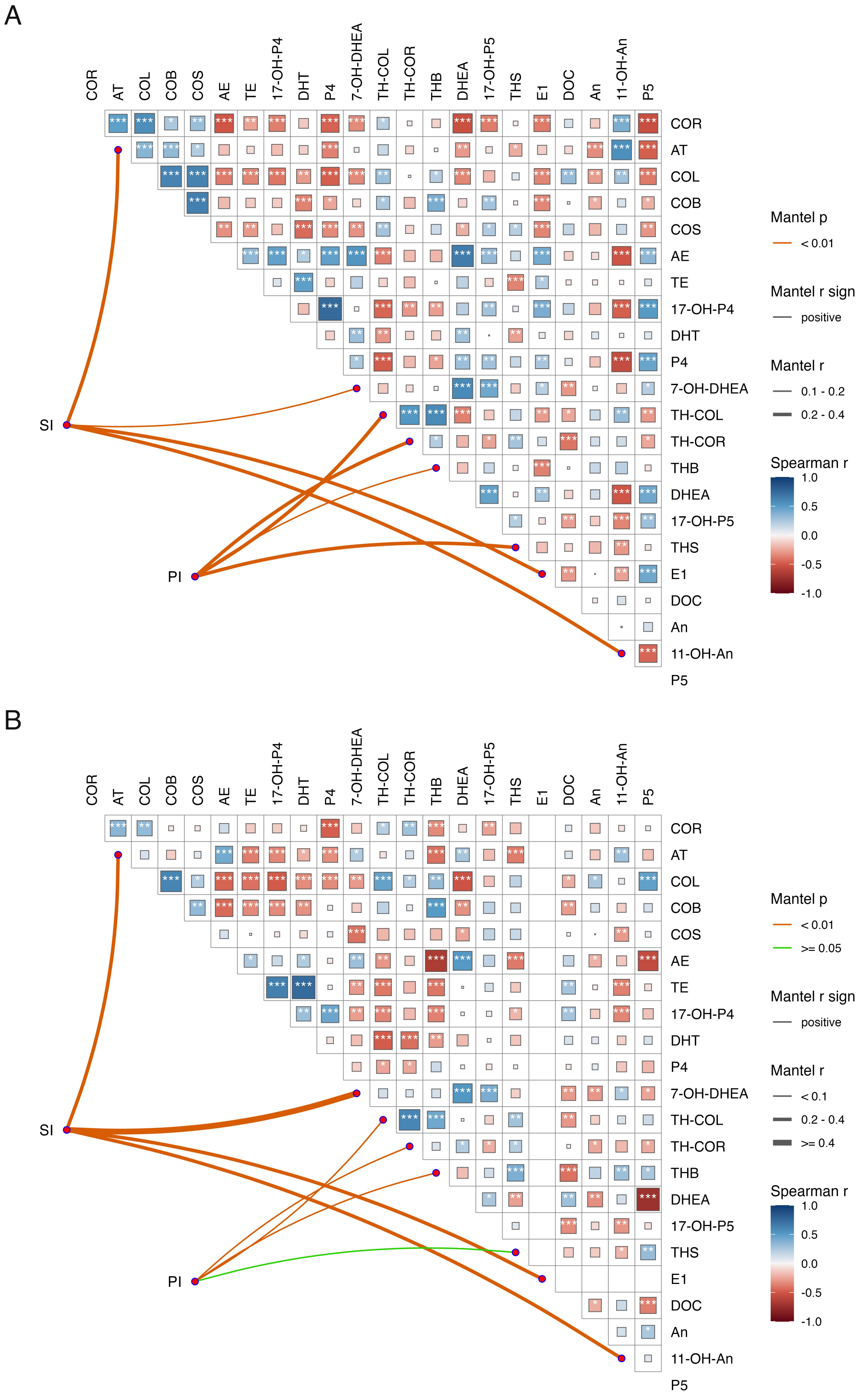
A
COR AT COL COB COS AE TE 17-OH-P4 DHT P4 7-OH-DHEA TH-COL TH-COR THB DHEA 17-OH-P5 THS E1 DOC An 11-OH-An P5
SI
PI
Mantel p
< 0.01
Mantel r sign
positive
Mantel r
0.1 - 0.2
0.2 - 0.4
Spearman r
1.0
0.5
0.0
-0.5
-1.0
B
COR AT COL COB COS AE TE 17-OH-P4 DHT P4 7-OH-DHEA TH-COL TH-COR THB DHEA 17-OH-P5 THS E1 DOC An 11-OH-An P5
SI
PI
Mantel p
< 0.01
>= 0.05
Mantel r sign
positive
Mantel r
< 0.1
0.2 - 0.4
>= 0.4
Spearman r
1.0
0.5
0.0
-0.5
-1.0

Fig. S7. Initial DNN edge weights based on steroid and CA correlations.

(**A**) Spearman correlation heatmaps displaying inter-steroid correlations, along with associated p-values, for (**A**) females and (**B**) males. Correlations between CA and eight steroids relevant to PI and SI are represented by connecting lines, with line weights indicating correlation strength and colors indicating significance levels (p-values). Statistical significance, blank, all zero values (non-test); * $P_{adj} < 0.05$; ** $P_{adj} < 0.01$; *** $P_{adj} < 0.001$.

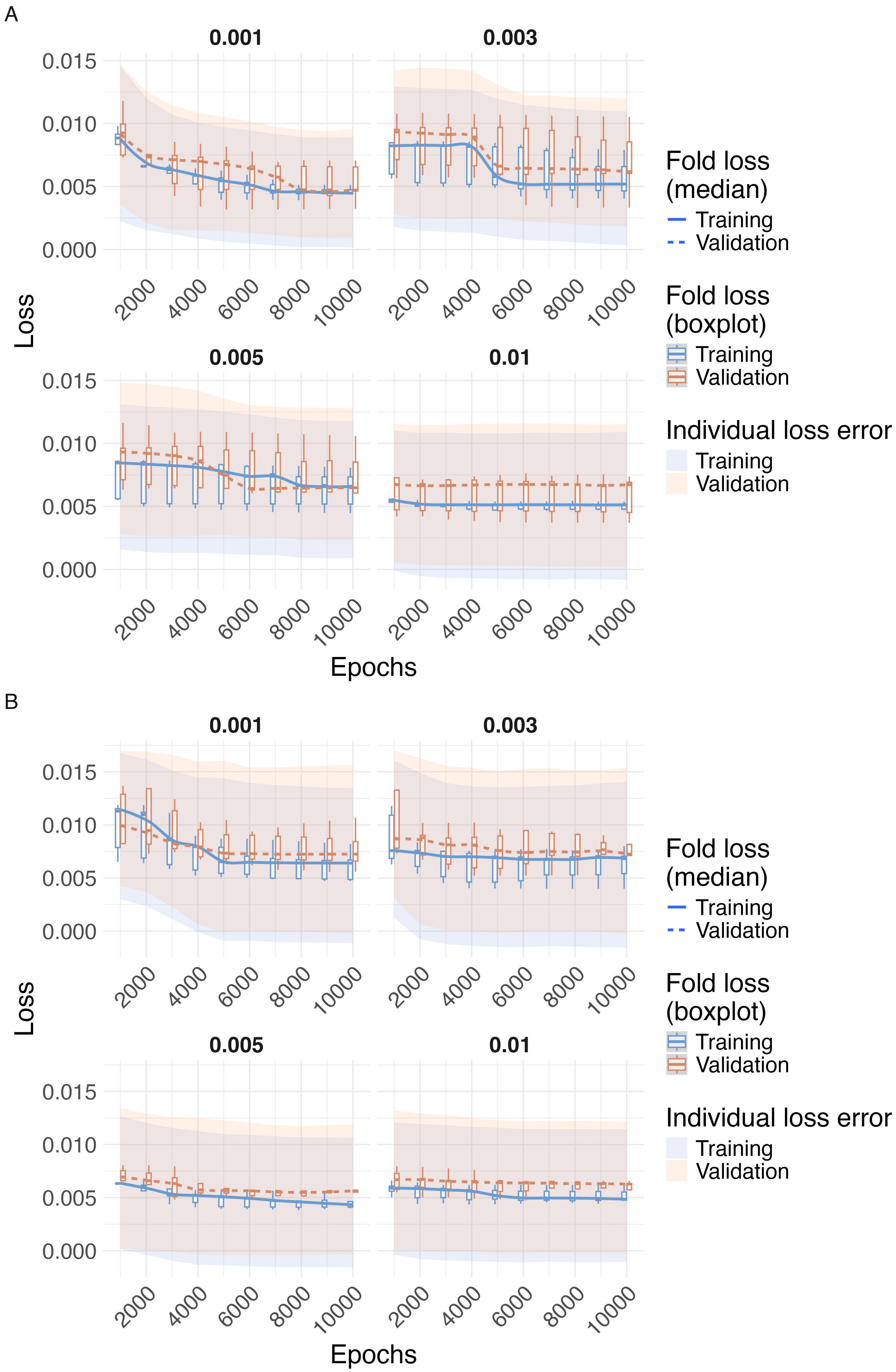
A
0.001
0.003
0.005
0.01
0.015
0.010
0.005
0.000
2000
4000
6000
8000
10000
Loss
Epochs
Fold loss (median)
Training
Validation
Fold loss (boxplot)
Training
Validation
Individual loss error
Training
Validation
B
0.001
0.003
0.005
0.01
0.015
0.010
0.005
0.000
2000
4000
6000
8000
10000
Loss
Epochs
Fold loss (median)
Training
Validation
Fold loss (boxplot)
Training
Validation
Individual loss error
Training
Validation

Fig. S8. Hyperparameter optimization through 5-fold cross-validation.

Model loss distributions across epochs for different learning rates in both (**A**) female and (**B**) male models. Each subplot represents model losses on the training (modeling) and validation folds at intervals of 1000 epochs, up to a maximum of 10000 epochs. The learning rate value for each subplot is indicated in the respective panel.

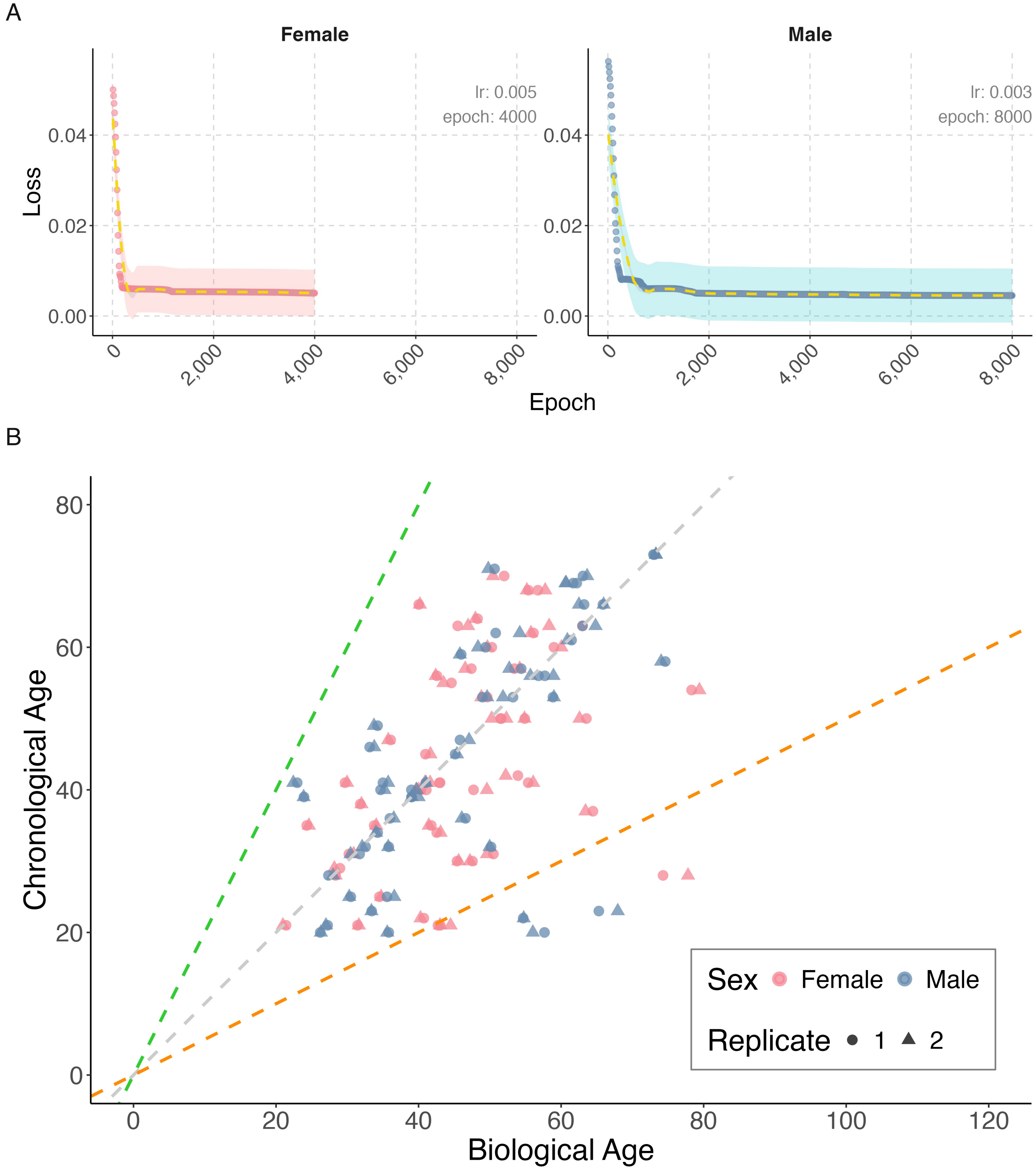

Fig. S9. Training performance of the DNN model with optimal hyperparameters.
(**A**) Training curves for females and males samples, illustrating performance metrics based on the corresponding optimal learning rates (lr) and epochs. Shaded areas represent the standard deviation of individual training losses. (**B**) Scatter plot comparing predicted BA against true CA for females (n = 98, including two replicates) and males (n = 98, including two replicates).

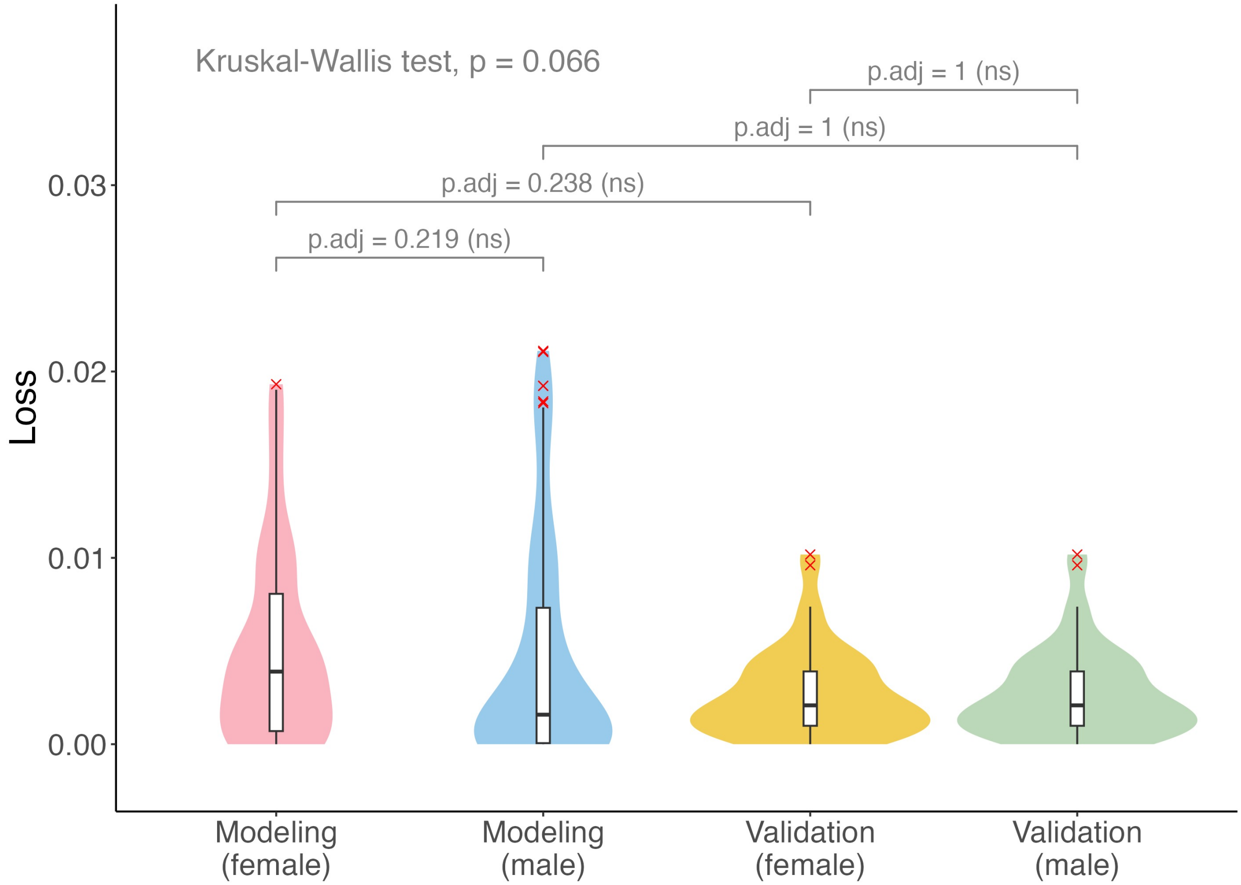

Fig. S10. Statistical analysis of Weighted Symmetric Arc-Tangent Loss (WSATL) values across all groups.

WSATL values were statistically analyzed across all groups using the Kruskal-Wallis test with Bonferroni correction for overall comparisons, and the Wilcoxon test with Bonferroni adjustment for pairwise comparisons between specific groups. The modeling dataset includes females (n = 98, with two replicates) and males (n = 98, with two replicates), while the validation dataset comprises females (n = 50, with two replicates) and males (n = 50, with two replicates). Statistical significance, ns, $P_{adj} \geq 0.05$.

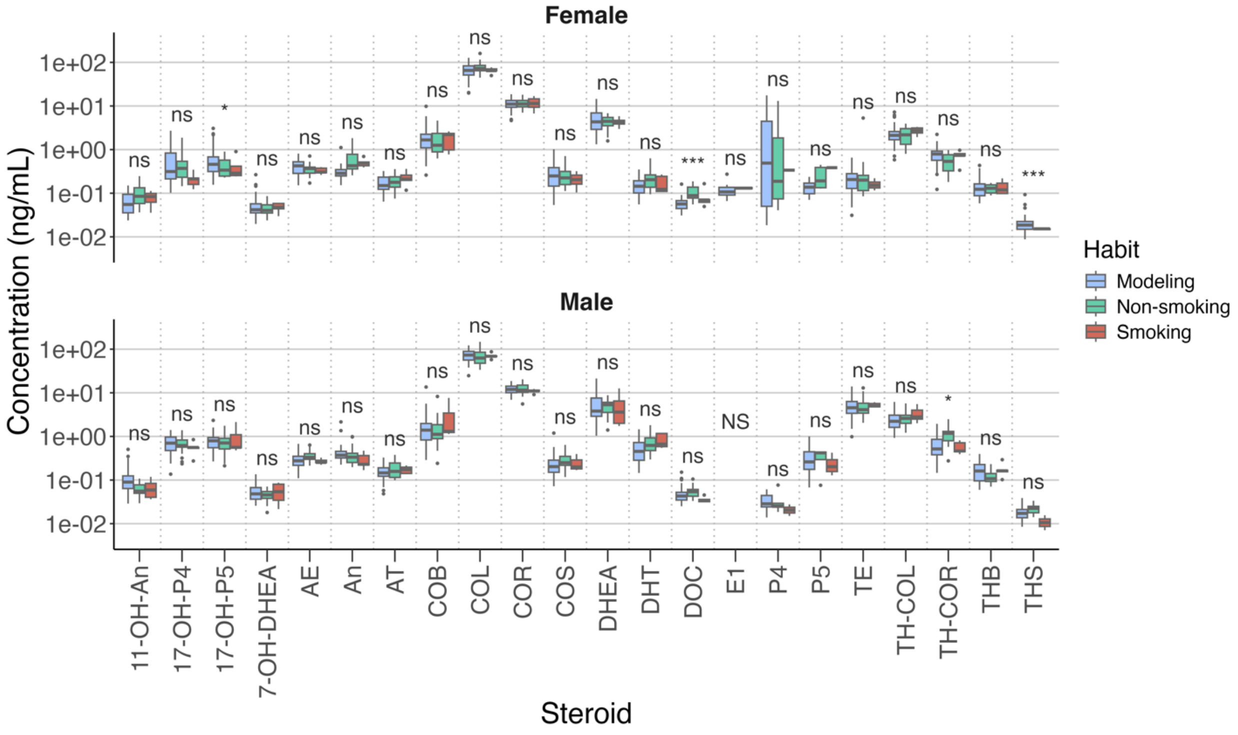

Fig. S11. Distribution of steroid concentrations before and after scaling for different smoking habit.

Univariate analysis for smoking habit, using the Kruskal-Wallis test adjusted by Bonferroni correction. The data for smoking habit include females (Modeling, habit unknown: n = 49; Non-smoking: n = 20; Smoking: n = 5) and males (Modeling, habit unknown: n = 49; Non-smoking: n = 20; Smoking: n = 5) from both the modeling and validation dataset. Statistical significance, NS, all zero values (non-test); ns, $P_{adj} \geq 0.05$; * $P_{adj} < 0.05$; ** $P_{adj} < 0.01$; *** $P_{adj} < 0.001$.

Table S1. Abbreviations and suppliers of steroid and internal standards.

Table S2. Parameters for the analysis of steroids by LC-MS/MS.

Table S3. Validation results for the developed LC-MS/MS method.

Table S4. Primary-derived data for BA modeling of all samples.

Table S5. Summary of reference steroid ranges described in the literature.

Table S6. Summary of quantitative analysis of steroids in human serum.

Table S7. Node and edge parameter results by sex in modeling.

Table S8. Post-hoc Tukey's HSD analysis results.

Table S9. Details of R environment and packages.
